# Analysis of the Subunit Stoichiometries in Viral Entry

**DOI:** 10.1371/journal.pone.0033441

**Published:** 2012-03-30

**Authors:** Carsten Magnus, Roland R. Regoes

**Affiliations:** Integrative Biology, The Swiss Federal Institute of Technology, Zurich, Switzerland; Albert Einstein College of Medicine, United States of America

## Abstract

Virions of the Human Immunodeficiency Virus (HIV) infect cells by first attaching with their surface spikes to the CD4 receptor on target cells. This leads to conformational changes in the viral spikes, enabling the virus to engage a coreceptor, commonly CCR5 or CXCR4, and consecutively to insert the fusion peptide into the cellular membrane. Finally, the viral and the cellular membranes fuse. The HIV spike is a trimer consisting of three identical heterodimers composed of the gp120 and gp41 envelope proteins. Each of the gp120 proteins in the trimer is capable of attaching to the CD4 receptor and the coreceptor, and each of the three gp41 units harbors a fusion domain. It is still under debate how many of the envelope subunits within a given trimer have to bind to the CD4 receptors and to the coreceptors, and how many gp41 protein fusion domains are required for fusion. These numbers are referred to as subunit stoichiometries. We present a mathematical framework for estimating these parameters individually by analyzing infectivity assays with pseudotyped viruses. We find that the number of spikes that are engaged in mediating cell entry and the distribution of the spike number play important roles for the estimation of the subunit stoichiometries. Our model framework also shows why it is important to subdivide the question of the number of functional subunits within one trimer into the three different subunit stoichiometries. In a second step, we extend our models to study whether the subunits within one trimer cooperate during receptor binding and fusion. As an example for how our models can be applied, we reanalyze a data set on subunit stoichiometries. We find that two envelope proteins have to engage with CD4-receptors and coreceptors and that two fusion proteins must be revealed within one trimer for viral entry. Our study is motivated by the mechanism of HIV entry but the experimental technique and the model framework can be extended to other viral systems as well.

## Introduction

To infect a cell, enveloped viruses must have a mechanism to attach to their target cells and to fuse their membrane with the target cell membrane. For this purpose the virions express spikes on their surface that are capable of binding to target cell receptors and after several conformational changes the spikes reveal fusion domains. Some viruses need low pH, others bind to several receptors for inducing the necessary rearrangements in the viral surface proteins for unmasking their fusion peptides [1].

The Human Immunodeficiency Virus (HIV) has trimers of the heterodimeric envelope proteins (Envs) gp120 and gp41 embedded in its surface [2]–[4]. These trimers first establish contact with CD4 receptors on the target cell [5]. This engagement leads to conformational changes in the envelope protein allowing a coreceptor, most commonly CCR5 or CXCR4, to bind [6]. A series of rearrangements in the viral envelope protein gp41 leads to the insertion of the fusion peptide in the cell membrane [1] and eventually fusion of the two membranes.

Recently, the structure of the trimers and the attachment sites were visualized by crystallization studies [7]–[11]. However, these studies cannot inform about quantitative aspects of viral entry that are commonly described by stoichiometric parameters. To estimate these parameters, infectivity experiments with pseudotyped virions in combination with mathematical models can be employed. The *stoichiometry of entry* is defined as the minimal number of trimer – cell receptor interactions needed for cell entry and was studied in [12]–[15]. The concept of entry stoichiometry is based on the fact that a virion has to get close enough to the cell membrane for insertion of the fusion protein. As the viral and the cellular membranes are both lipid bilayers they repel each other. To overcome this repellent force, spikes must attach to cellular receptors and pull the membranes closer together. We assume here that there is a critical number of spikes that have to work together to ensure that the two membranes get close enough. If there are fewer trimer - receptor interactions than this number, the two membranes will not fuse. However, this number is only indirectly related to the probability with which a virion can infect a cell. The infection probability is 0 when the number of spikes is smaller than the critical number, and larger than 0 if the this number is above the critical number. But the exact value of the infection probability is a compound quantity that involves many processes and their probabilities, such as the spatio-temporal dynamics of virions in the cell culture, the fusion of the virion and cell membrane, and the processes leading up to the integration of the genetic material into the host cell genome.

The *stoichiometry of (trimer) neutralization* is the minimal number of monoclonal antibodies needed to neutralize one single trimer [16], [17]. By combining the stoichiometry of neutralization with the stoichiometry of entry, one can calculate the number of antibodies needed to neutralize a virion and a whole population [18].

**Figure 1:**
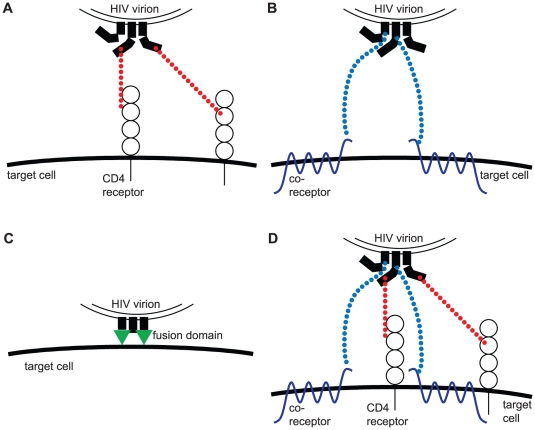
Sketch of the different subunit stoichiometries. (A) CD4 subunit stoichiometry: The number of envelope-CD4 receptor interactions (red dotted lines) one trimer requires for taking part in mediating cell entry. (B) Coreceptor subunit stoichiometry: The number of envelope-coreceptor interactions (blue dotted line) one trimer requires for taking part in mediating cell entry. (C) Fusion subunit stoichiometry: The number of fusion proteins (green triangles) that have to be exposed such that the trimer takes part in mediating cell entry. (D) Relative location of functions: Do two functions (in this sketch CD4 and coreceptor binding) have to be located on the same envelope protein within one trimer, for taking part in cell entry? Here one of the two coreceptors binds to one envelope protein to which no CD4 receptor is bound.

In addition to these stoichiometric parameters, Yang et al. [19] defined the number of subunits within an HIV-1 envelope glycoprotein trimer that is generally required such that this trimer takes part in mediating viral entry as the *subunit stoichiometry*. In their analysis they do not distinguish between CD4 binding, coreceptor binding or revealing the fusion protein. However, it is possible that a different number of coreceptors must bind to the trimer than CD4 proteins or a different number of fusion proteins are needed to mediate cell entry than receptors must bind. Indeed, previously obtained data might indicate these differences [19]. Therefore, it is necessary to study quantitative aspects for every step involved in viral entry separately. To capture these differences, we refine the subunit stoichiometry by defining three stoichiometric parameters:

1. the *CD4 subunit stoichiometry*, i.e. the number of envelope protein – CD4 interactions within one trimer required for taking part in viral entry (Figure 1A)
2. the *coreceptor subunit stoichiometry*, i.e. the number of envelope protein – coreceptor interactions within one trimer required for taking part in viral entry (Figure 1B)
3. the *fusion subunit stoichiometry*, i.e. the number of fusion proteins per trimer that have to be exposed for taking part in membrane fusion (Figure 1C)

In short, the strategy to infer these stoichiometries is the following: Infectivity assays with pseudo-typed virions expressing heterotrimers of HIV-1 envelope proteins with wild-type proteins and proteins deficient in receptor binding are the basis of the presented framework [13], [16], [20]. One can address different stoichiometric parameters by including different mutations in the envelope protein. A sophisticated mathematical model for the infectivity experiments has to be formulated because the stoichiometric parameters cannot be read out directly from these experiments. In this paper we show, how the mathematical framework for the stoichiometry of entry and trimer neutralization derived in [15], [17] can be extended to study subunit stoichiometries. We also show which type of mutations in the envelope proteins of the pseudo-typed viral stocks should be used to estimate each of the stoichiometric parameters. As an example of how our models can be applied, we re-analyze a previously published data set [19] with our models.

By subdividing the subunit stoichiometry into numerical requirements for the different steps involved in viral entry, new questions arise: Do the CD4 receptor and the coreceptor bind to different subunits or do they bind to the same subunit within one trimer? In addition, one can ask whether the fusion proteins of those subunits that were bound to the receptors are involved in the final fusion process. Studies on *monomeric* envelope proteins showed that the coreceptor can only bind after the CD4 receptor has bound [21]. Therefore CD4 is the primary receptor for HIV-1. A cascade of conformational changes induced by CD4 binding allow coreceptor binding, which is followed by the insertion of the viral fusion protein into the cellular membrane [22]. However, Salzwedel and Berger [23] hypothesized that not each subunit in the *trimer* has to be able to perform all these functions. Liu et al. [24] even show that virions expressing mixed trimers consisting of CD4-binding deficient envelope proteins and envelope proteins with inactivated fusion proteins can still infect cells *in vitro*. Therefore, it is possible that the subunits of one trimer divide the different tasks involved in infection among themselves. Given two functions of the envelope protein (these might be the two kinds of receptor binding or the fusion protein), we define *subunit cooperation* with respect to these functions as the ability that these functions are located on different envelope proteins within one trimer. The experimental systems as well as the mathematical models depend on the actual values of the different subunit stoichiometries. Because these values still have to be determined, we subdivide the study of the subunit cooperation into three cases. Each case describes one of the possible combination of two subunit stoichiometries for which subunit cooperation is possible. This theoretic framework can also be extended to study other viral systems.

The stoichiometry of entry and neutralization refine the understanding of viral neutralization by antibodies and can therefore help in rational vaccine design [12]–[17]. The subunit stoichiometries will not only inform about the structural requirements on the virus for being infectious but also inform about the structural requirements on a host cell for being infectable. The concept of subunit stoichiometries will help to design and dose entry inhibitors directed against viral spikes such that the sufficient number of subunit engagements are disturbed. Once the subunit stoichiometries are determined, it will be possible to predict how many receptors a target cell must express for being infectable. Entry inhibitors that are directed against host cell receptors can then be designed more rationally to lower the probability that a virion infects the cell. In addition to these practical applications, knowing the subunit stoichiometries and how the functions must be distributed within one trimer will help to answer the question of why HIV expresses spikes consisting of three identical subunits. If only two CD4-envelope and two coreceptor- envelope engagements as well as two fusion proteins were needed such that the trimer can take part in mediating cell entry, an envelope-dimer would have also been sufficient for viral entry.

## Methods

### Experimental setup

Here, we briefly describe the experimental setup for the determination of the subunit stoichiometries. The basic concepts behind these experiments are very similar to those for studying the stoichiometry of entry and neutralization [13], [15], [16] and are explained in [19] in more detail.

To estimate the subunit stoichiometries and to resolve the subunit cooperation, a series of infectivity assays with pseudo-typed virions have to be performed. The virions must be genetically engineered such that they report the infection but do not replicate. For these assays, the virions are produced by transfecting virus producer cells with a set of plasmids. One plasmid provides all the genetic information to assemble infectious but replication-incompetent virions with the exception of the viral envelope. This protein is provided on another plasmid. Mixed envelope proteins are expressed when mixing wild-type envelope encoding plasmids and envelope encoding plasmids carrying a mutation in the region of interest for the question to be studied. The mutant envelopes should harbor only one (or few) amino acid changes compared to the wild-type such that they can form functional hetero-trimers [25]. Different viral stocks with varying fractions of mutant envelope encoding plasmids are produced. The more mutant envelope proteins are mixed to one viral stock, the fewer functional trimers are expressed on the virus surface and the fewer virions infect cells. The infectivity of these viral stocks is measured via the expressed luciferase and is proportional to the number of virions that successfully infected a cell.

The different stoichiometric parameters defined in the Introduction can be addressed by using different mutations in the envelope proteins. If, for example, one wants to study the number of CD4-envelope bonds within one trimer, the mutated envelope protein must carry a mutation that renders the envelope incapable to bind to CD4. Table 1 summarizes the stoichiometric questions and the mutations that have to be used in the corresponding experiments.

**Table 1:** Overview of the different stoichiometric parameters and the mutations in the envelope protein, that might be used to study these parameters.

parameter	Definition	mutation
CD4 subunit stoichiometry	number of envelope protein – CD4 interactions within one trimer required for viral entry	envelope deficient of CD4 binding, e.g. [19]
coreceptor subunit stoichiometry	number of envelope protein – coreceptor interactions within one trimer for viral entry	envelope deficient of coreceptor binding, e.g. [19]
fusion subunit stoichiometry	number of fusion proteins per trimer that have to be exposed for membrane fusion	envelope with a non-functional fusion protein unit, e.g. [19]
subunit cooperation	Do two functions A and B (e.g. receptor binding or revelation of the fusion domain) have to be located on two different subunits?	(i) envelope proteins defective of the two functions simultaneously; (ii) two different mutants: each defective of one of the two functions

### Mathematical models

Our models predict the relative infectivity of a pseudotyped virus stock as a function of the fraction of mutated envelope proteins. They account for the fact that virions express more than one trimer on the surface and that this number can vary from virion to virion [26]. Therefore we assume the trimer number on each virion to be drawn from the trimer number distribution 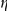. In addition, we assume that the envelope proteins within a cell reflect the composition of envelope encoding plasmids used for generating the viral stock and that trimers form perfectly randomly, mathematically speaking according to a Binomial distribution, out of this envelope pool. Further we assume that the receptor density on the target cells is sufficiently high to bind every functional binding region. A trimer is counted as functional when the number of receptor-envelope interactions is at least as big as the subunit stoichiometry and the number of fusion proteins at least as big as the fusion subunit stoichiometry. A virion is counted as infectious when the number of functional trimers is at least as big as the stoichiometry of entry 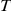 [15].

Basically, we can differentiate two kinds of models on the level of the experimental requirements: models for systems with wildtype and one mutant envelope proteins and models for systems with wildtype and two mutations either on the same or two different envelope proteins. The first kind of models allow us to study the subunit stoichiometries. The second kind of model systems allow us to study the subunit cooperation. We classify our models according to this categorization. All parameters used in the models and their definitions are listed in table 2.

**Table 2:** Parameter definitions.

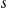	number of trimers on virion
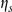	probability that a virion has 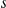 trimers
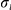	subunit stoichiometry for the receptor 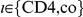
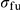	subunit stoichiometry of fusion
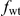	fraction of plasmids encoding for wildtype envelope proteins
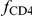	fraction of plasmids encoding for CD4 binding deficient envelope proteins
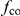	fraction of plasmids encoding for coreceptor binding deficient envelope proteins
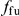	fraction of plasmids encoding for fusion-incompetent envelope proteins
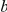	variable indicating whether envelope proteins cooperate with respect to two
	functions A and B ( 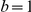 ) or not ( 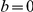 )
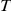	stoichiometry of entry

#### Models with one mutation

Pseudotyped virions harboring mixtures of wildtype envelope proteins and one type of mutant envelope proteins can be used in infectivity experiments to study subunit stoichiometries. Depending on the mutation, a one-mutation system informs about the number of CD4-envelope bonds, 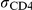, or coreceptor-envelope bonds, 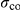 within one trimer that a trimer requires for taking part in mediating viral entry. Furthermore, the one-mutation system can inform about the number of fusion proteins within one trimer, 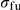, that have to engage with the cellular surface such that the trimer takes part in mediating viral entry. All three stoichiometric parameters can be 1, 2 or 3 (in Figure 1A–C we illustrate the stoichiometries for the case 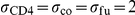 ).

**Figure 2:**
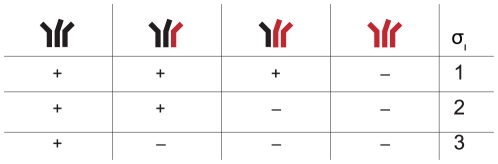
Dependence of the trimer's functionality on the subunit stoichiometry 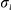
 **.** Wildtype envelope proteins are colored black and mutant envelope proteins are colored red. Functional trimers are marked with “+” and non-functional trimers with “−”.

For the models, it is not important which mutation prevents one envelope protein from engaging in the fusion process. Therefore, we present one model that can be used for estimating all three stoichiometric parameters. Let 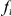 be the fraction of envelope encoding plasmids with a mutation making CD4-binding (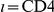) or coreceptor-binding (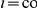) impossible respectively disrupting the fusion protein (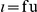). In the experiments the trimer's functionality depends on the number of mutations within one trimer and the actual stoichiometric parameter. Figure 2 shows which combinations of envelope proteins are functional for the possible values of the stoichiometries 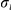.

Let 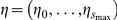 be the distribution of trimer numbers, i.e. the probability that a virion has 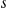 trimers is 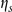 for 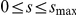, and 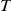 the stoichiometry of entry, i.e. the minimal number of trimers required for mediating entry (as defined in [15]). To produce a virus stock, plasmid encoding for the genetic information of the virus as well as plasmids encoding for the different envelope proteins are mixed, and used to transfect virus producer cells. These plasmids are translated within the cell, and the translated envelope proteins form trimers that are transported to the viral surface. Let the fraction of plasmids encoding mutated envelopes be 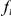. We assume that envelope proteins trimerize perfectly randomly, which means that the probability of a mutant envelope protein to be recruited into a trimer is only dependent on its frequency among all envelope proteins in the virus producer cell. The probability that a trimer has 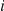 mutants is then
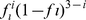
(1)A trimer takes part in mediating cell entry if at least 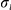 subunits bind to the corresponding receptor. Therefore, a trimer is functional when it has no more than 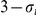 mutated envelope proteins. The probability for a trimer to be functional, 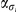, is
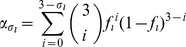
(2)Given a virion with 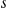 spikes on its surface and the stoichiometry of entry 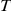, the virion is able to infect a cell when it has 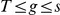 functional spikes. This means that the probability that a virion with 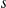 spikes is infectious can be calculated by summing the probabilities that a virion with 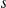 trimers has exactly 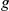 functional ones:
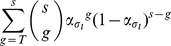
(3)According to our definition of the trimer number distribution, 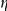, a virion has 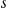 virions with the probability 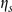. Using the probability that a virion has 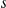 trimers, we can calculate the relative infectivity 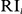 of a viral stock with a fraction of 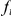 mutated envelope proteins as the weighted sum of the probability that a virion with 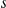 trimers is functional. Experimentally the infectivity of a pseudotyped viral stock is compared with the infectivity of a wildtype viral stock. Therefore, the relative infectivity has to be scaled with the probability that a wildtype virion is infective and we obtain
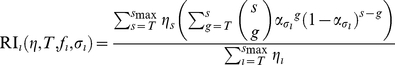
(4)


#### Models with two mutations

There are three important steps involved in trimer mediated membrane fusion in HIV-1 entry that are subject of our subunit stoichiometric studies: CD4-binding, coreceptor binding and revelation of the fusion protein. To study whether the CD4 receptor and the coreceptor must bind to the same envelope subunit and whether the fusion protein must also be revealed in this subunit, only two functions should be tested in the same experimental system. This strategy minimizes confounding side effects arising by genetically engineering the envelope protein trimers. Three questions can then be addressed: (i) Do the CD4-receptor and the co-receptor bind to the same subunit? (ii) Does CD4 binding lead to the revelation of the fusion domain of the bound subunit, or another subunit within the trimer? (iii) Does coreceptor binding lead to the revelation of the fusion domain of the bound subunit, or another subunit within the trimer?

**Figure 3:**
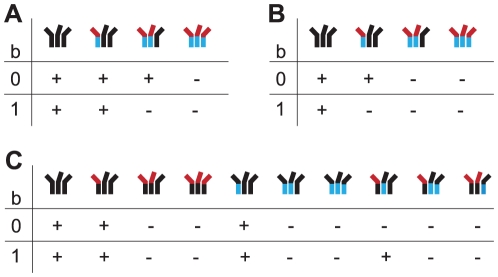
Overview of the functional trimers (marked with “+”) for different experimental setups for studying the location of functional units within one trimer. Mutations making the envelope protein defective for function A are colored red and those making the envelope defective for function B are colored blue. In the cases 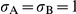 (A) and 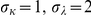 (B), wildtype and envelope proteins defective of function A and B (double mutants) have to be used. In the case 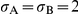 (C) wildtype, A-defective and B-defective envelope proteins have to be used in the infectivity assays with pseudotyped virions. The first row in each table correspond to a scenario in which the two functional units A and B must be located on the same envelope protein (no cooperation, 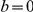). The second row correspond to a scenario in which the functional units have to be located on different envelope proteins (cooperation, 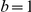).

We define *subunit cooperation* in a more general context. Assume an enveloped virion with spikes on its surface that are engaged in mediating cell entry. Each spike has three identical subunits. Each subunit fulfills different functions required for cell entry. We denote the single subunit stoichiometries of two of these functions A and B with 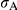 and 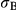, respectively, and assume that the actual values are known. Loosely speaking, no cooperation happens when the functions A and B are located on the same protomer and the two functions cooperate when they are located on different protomers. The exact definition of cooperation depends on the actual values of the single subunit stoichiometries. For 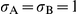 and 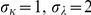, for 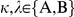 cooperation is defined in the sense that the different functions must be located on different protomers. For 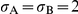 cooperation means that there is one protomer bound to A and B, one protomer only to A and the third protomer only to B. To study whether the two functions A and B cooperate, infectivity assays with pseudotyped virions expressing wild-type and mutated envelope proteins with two mutations must be employed. The mutations can be either present on the same envelope protein or on different proteins. Which envelope protein mutants should be used and which mathematical models have to be applied to address this question, depends on the values of the subunit stoichiometries 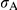 and 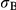. If one of the functions has subunit stoichiometry 3, the second functional unit must be located on an envelope protein that is already engaged, independently of its subunit stoichiometry. If none of the subunit stoichiometries is 3, we have to distinguish the following cases as in the definition of subunit cooperation:
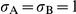



In the case of both subunit stoichiometries being one, there are two possibilities how the functional units A and B can be distributed over the three envelope proteins of the trimer. Either the two functions A and B are located on the same subunit within one trimer (no cooperation) or on different ones, i.e. different subunits cooperate and divide the functions among themselves. To address this question, the infectivity of pseudotyped virions must be measured that express mixed trimers of wildtype envelope proteins and mutant envelope proteins being defective of both functions A and B. In a scenario in which both functional units are located on different envelope proteins, trimers with two double-mutant envelope proteins are not functional. In contrast, these trimers are functional in a situation in which both functions are located on the same envelope protein (see Figure 3 (A)).

For modeling these scenarios, we make predictions for the relative infectivity RI for varying fractions of double mutant Env encoding plasmids. Let 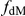 be the fraction of envelope encoding plasmids with the double mutation. Within the transfected virus producer cell, these plasmids will be translated into envelope proteins being defective of both functions at the same time. Let 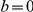 denote the scenario in which both functional units are present on the same envelope protein (no cooperation) and 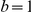 denotes the scenario in which the two functions are divided between two different envelope proteins (subunit cooperation). The probability that a trimer is functional depends on the number of double mutants and the mode of cooperation:
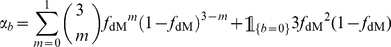
(5)To calculate the relative infectivity, the probability of forming a functional trimer in equation 4 has to be replaced by equation 5:
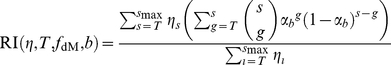
(6)


### 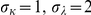 for 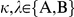


In this scenario, one of the two functional units A or B has subunit stoichiometry one and the other subunit stoichiometry two. There are two possibilities how the three functions can be distributed over the trimer. Either one 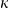-unit is present at one of the envelope proteins with the functional 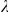-unit (no cooperation), denoted by 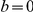, or the 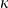-unit is present on an envelope protein without any of the two 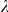-units (cooperation), denoted by 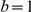. Figure 3 (B) shows all possible trimer combinations and their functionality depending on the two scenarios. By adapting the probability to form a functional trimer, we obtain for the relative infectivity:
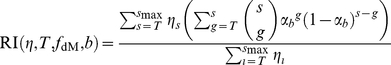
(7)with the probability to from a functional trimer in this scenario:
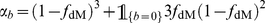
(8)

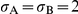



If both subunit stoichiometries are two, there are two possible scenarios how the four functions can be distributed over the three envelope proteins of one trimer. As there is only one site for function A and one site for function B per envelope protein, either both functions have to be located on the same two envelope proteins (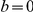, no cooperation) or one of the trimer has the two functions A and B and the other two envelope proteins have either function (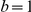, cooperation). An experimental setup with a mixture of wildtype envelope proteins and two single mutant envelope proteins allows us to study this question. One envelope protein mutant must harbor a defect in the functional unit A. The other mutated envelope protein must render this envelope protein defective for function B. In total, there are three different envelope proteins in the envelope pool within the virus producer cell. 10 different trimers can form. Figure 3 (C) shows these envelope combinations and their functionality in the cooperation as well as the non-cooperation scenario.

Let 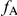 be the fraction of plasmids encoding for envelope proteins that are A-defective and 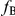 the fraction of plasmids encoding for B-defective envelope proteins. Note that 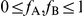 and the fraction of wildtype envelope encoding plasmids, 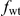, simply is 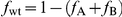. Hence, the relative infectivity in this scenario is
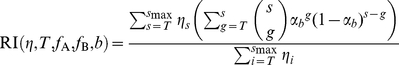
(9)where
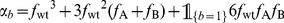
(10)is the probability that a trimer is functional.

## Results

### Identifying subunit stoichiometries

In the Model section we derived the relative infectivity of pseudotyped viral stocks expressing mixed trimers for estimating the CD4 subunit stoichiometry, the coreceptor subunit stoichiometry and the fusion subunit stoichiometry. The model (equation 4) stays the same for any subunit stoichiometric estimation. Only the viral stocks for the infectivity assays differ in the corresponding envelope mutation. Therefore, we show the properties of the model for estimating subunit stoichiometries generically without specifying one particular subunit stoichiometry.

**Figure 4:**
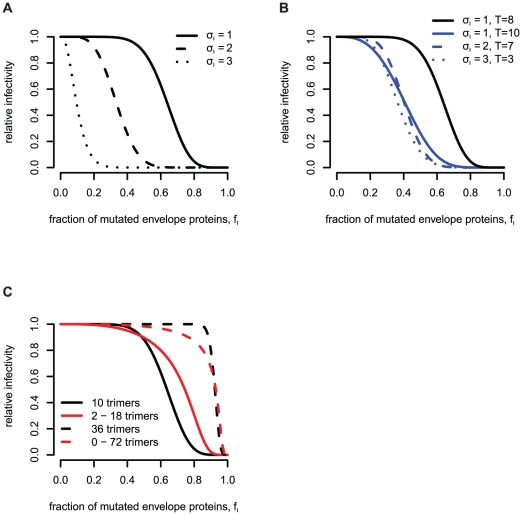
Predictions for the relative infectivity for different subunit stoichiometries in the basic model (**equation 4**). For plot (A) and (B) we assume that virions have exactly 10 trimers. For plot (A) and (C) we fix the stoichiometry of entry at 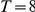, according to our estimate in [15]. (A) Influence of the subunit stoichiometry 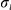 on the predictions for the relative infectivity. (B) Higher stoichiometries of entry shift the RI curve to the left (solid curves). Together with the effect of the subunit stoichiometry one can find parameter combinations of 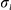 and 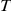 that lead to similar predictions (blue curves). (C) Dependence of the relative infectivity on the trimer number distribution.

The relative infectivities for the subunit stoichiometry 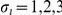, as functions of the fraction 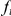 of envelope proteins with a mutation are predicted to be sigmoid curves. The smaller the subunit stoichiometry is, the higher the fraction of mutated envelope proteins, 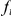, must become to decrease the relative infectivity (Figure 4A). In this Figure, the number of trimers on virions is fixed to 10 (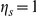 for 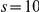, 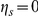 else) and the stoichiometry of entry 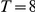.

In Figure 4B we show the effect of the stoichiometry of entry 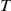, on the predictions of the relative infectivity. The higher the stoichiometry of entry is, the smaller must be the fraction of mutated envelope proteins for a decrease in the relative infectivity (solid blue and black lines). By increasing the subunit stoichiometry, 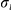 and simultaneously decreasing the stoichiometry of entry, 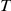, the predictions for the relative infectivity can become very similar (blue curves in Figure 4B). Therefore it is important to first determine the stoichiometry of entry according to [15] before being able to estimate the subunit stoichiometry. In this Figure, the trimer numbers on virions are fixed to 10.

The trimer number distribution, 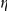, also influence the predictions of the relative infectivity. The higher the mean number of trimers on the virions, the higher must the fraction of mutated envelope proteins be to observe a decrease in relative infectivity. Figure 4C in which the mean number of trimers is 10 (solid curves) and 36 (dashed curves), respectively, shows this effect. The predictions for the relative infectivity become smoother for increasing variance. The variances for the black curves in Figure 4C are 0 and for the red curves the variances are 24 (solid red curve) and 444 (dashed red curve). The subunit stoichiometry in Figure 4C is set to 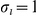 and the stoichiometry of entry 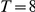.

Figure 4 shows, that the trimer number distribution and the stoichiometry of entry have important effects on the predictions of the relative infectivity and as a consequence on estimating the subunit stoichiometries. Therefore it is necessary to determine these quantities before estimating the subunit stoichiometries (as described in [15]). Zhu et al. [26] investigated trimers on HIV-1 virions and found a mean trimer number of 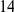 with variance 49. However, they observed only 40 virions. This small sample size is not sufficient to extrapolate the empirical trimer number distribution as a valid approximation of the real trimer number distribution. Instead, we use a discretized 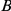-distribution with mean 14 and variance 7 [15], [17] as a trimer number distribution for the following figures and for the estimates in the section “Example: re-analysis of data of Yang et al. [19]”. In [15] we reanalyzed a data set by Yang et al. [13] with our models for the stoichiometry of entry and obtained 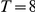 for our basic model. But we also showed that there is considerable uncertainty in this estimate originating in very stringent assumptions made in the basic model. Despite the uncertainties in the estimate of the stoichiometry of entry we use this value for all estimations of the subunit stoichiometries in the following sections to demonstrate the methods we provide in this paper. The resulting estimates should be taken with care and may need to be revised once better estimates of the stoichiometry of entry are available.

### Do envelope proteins cooperate within one trimer?

To identify whether e.g. the CD4 receptor and the coreceptor bind to the same envelope protein within one trimer, we introduced the more general concept of subunit cooperation. This framework can generally be used to study viruses expressing envelope proteins that have to bind to more than one receptor and/or carry a fusion domain on the envelope protein. The subunit stoichiometries of the different functions must be estimated first with the model framework presented above on the basis of infectivity experiments with pseudotyped virions. Once the stoichiometries are determined one can study whether the envelope protomers of one spike have to cooperate for the spike to be functional. Cooperation can only occur when the subunit stoichiometries of two studied functions are both less than the number of envelope proteins per viral spike. Here we developed a framework for trimeric viral spikes as they are expressed on HIV-virions. The experimental setup to determine subunit cooperation as well as the model framework is dependent on the actual values of the subunit stoichiometries of the two functions, denoted with 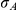 and 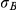. These stoichiometric parameters have to be determined before the mode of subunit cooperation can be identified. In the case 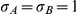 and in the case 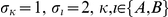, similar experimental setups must be used. The case 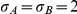 requires a more advanced experimental setup. In any case, trimer tables inform about whether a trimer is functional or not and are an easy tool to understand the model equations. In the following we describe the results for the case 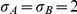 separately from the two other cases.

The models are based on the assumption that every potential binding site will be bound by the corresponding receptor. This can be guaranteed by using a target cell line with high receptor densities. Virions with a number of functional trimers exceeding the number of functional trimers needed for cell entry are assumed to end up infecting a cell with a certain probability. This probability cancels out in the expressions for the relative infectivity (equations 4, 6, 7, 9) because we compare the infectivity of pseudotyped virus stocks with the infectivity of a wild-type stock. This is why our models do not inform about the order with which the receptors bind to the protomers. However, for HIV there is experimental evidence that CD4 receptors have to bind first, followed by the coreceptor which induce the revelation of the fusion protein [21], [22].

### 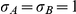 or 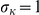, for at least one 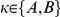


If at least one of the two subunit stoichiometries of the functional units A or B is one, infectivity assays with different pseudotyped virus stocks expressing wildtype and mutated envelope proteins have to be performed. The mutated envelope protein must have mutations in the regions of the functional units A and B such that both functional units in this mutated envelope protein are defective. Differently mixed trimers have different functionalities (Figure 3 (A) and (B)). The predictions for the relative infectivity in the different scenarios are based on the different functionalities of the trimers with wildtype and mutant envelope subunits.

**Figure 5:**
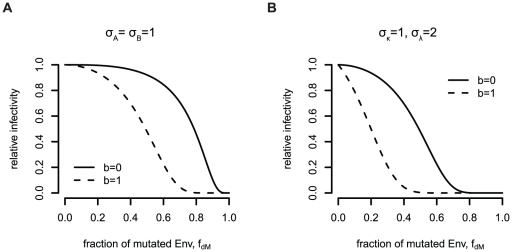
Predictions for the relative infectivity for different subunit stoichiometries using a double mutant that is defective of both function A and B for the single subunit stoichiometries (A) 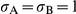
 **(corresponding to the trimer table in** **figure 3 (A)) and (B)** **** 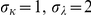 **for** 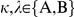 **(corresponding to the trimer table in** **figure 3 (B)** **).**

Figure 5 shows the predictions for the relative infectivity in a system with two mutations on the same envelope subunit. In Figure 5(A), the two subunit stoichiometries of the functional units A and B are both 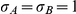. The solid curve shows the predictions for a scenario in which both functional units have to be located on the same envelope protein and the dashed curve shows the predictions of the relative infectivity for a scenario in which the two functional units can also be located on different envelope subunits.

In Figure 5(B) we show the predictions for the relative infectivity for the scenario in which one of the functional units has subunit stoichiometry 1 and the other subunit has stoichiometry 2. If the functional units must be located on different subunits, only wildtype homotrimers are functional trimers (second row in Figure 3(B)). The predictions for the relative infectivity in this scenario is shown by the dashed curve in Figure 5(B). If the functional unit with the 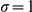-stoichiometry has to be located on an envelope protein with a functional unit of the 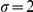-stoichiometry, the predictions for the relative infectivity look different (solid curve in Figure 5(B)). For the predictions of the relative infectivity in Figure 5 we assumed the stoichiometry of entry to be 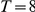 and as trimer number distribution we assumed the discretized B-distribution with mean 14 and variance 49 (in accordance to [15]).
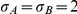



If both functional units have subunit stoichiometries two, an experimental setup with wildtype and envelope proteins simultaneously defective of A and B would not allow to dissect subunit cooperation. Instead pseudo-typed virions expressing trimers with wildtype envelope proteins as well as envelope proteins with mutations making the functional unit A defective and mutated envelope proteins with a defect in the functional unit B are required. By mixing three different envelope proteins, ten different trimers can be distinguished (Figure 3(C)). Two scenarios are possible: (b = 0) both functional units have to be located on the same two envelope proteins, (b = 1) one envelope protein in the trimer has the functional unit A and B, the two other envelope proteins have a different functional unit each.

**Figure 6:**
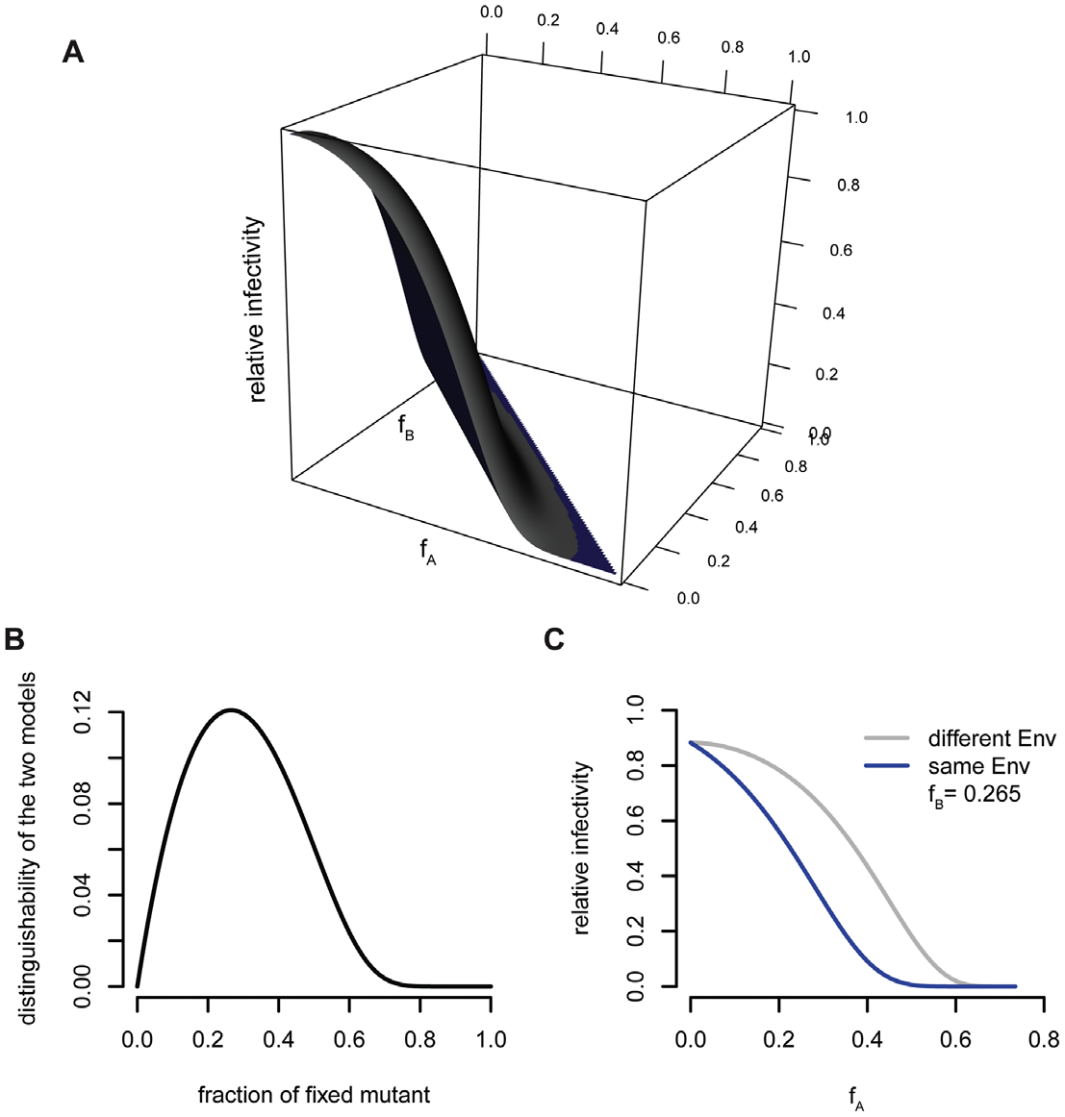
Predictions for the relative infectivity for 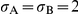
 **.** (A) The relative infectivity is shown as a function of the fractions of envelope proteins defective of function A, 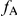, and the fraction of envelope proteins defective of function B, 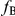. The grey plane shows the predictions for a model in which the two functional units A and B are located on the same envelope protein, the blue plane the model in which these function can be located on different envelope proteins. (B) The difference between the two models is the highest, if the fraction of one mutant is fixed at 0.265. (C) The relative infectivity for an experimental system in which the fraction of one mutant is fixed at maximum distinguishability and the fraction of the other mutant envelope protein is varied.

One now can predict the relative infectivities as a function of the fraction of A defective envelope protein, 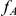 and B defective envelope protein, 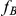. This means that one obtains relative infectivity planes instead of relative infectivity curves. In Figure 6(A) we show these predictions. The blue plane is the prediction for a scenario in which the two functional units must be located on the same envelope protein (b = 0, no cooperation) and the grey plane is the prediction for the cooperation scenario (b = 1). The relative infectivity planes differ for fixed values of the fraction of one of the mutants. Figure 6(B) shows the distance between the planes for the two cooperation scenarios as a function of the fraction of the fixed mutant. This distance is a measure for the distinguishability of the two scenarios. The maximal distinguishability is reached when fixing one mutant at a value of 0.265. The predictions for the two different scenarios are shown in Figure 6(C). This means that it is not necessary to determine the entire relative infectivity planes. To determine the entire planes would require 66 different viral stocks for a sufficiently high resolution. Instead, 8 different viral stocks with one fraction of mutant envelope encoding plasmids being fixed at 0.265 and the other varying between 0 and 0.735 will suffice. These predictions are made under the assumption of a discretized 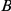-distribution with mean 14 and variance 49 for the trimer number distribution and the stoichiometry of entry 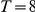 and may vary for other parameters, and may have to be revised if these input parameters change.

### Example: re-analysis of data of Yang et al. [19]

Yang et al. [19] studied the subunit stoichiometry with a combination of infectivity experiments and models for HIV-1. Their model did not account for the fact that virions have a variable number of trimers on their surfaces. Zhu et al [26] counted the number of trimers on 40 virions and found in the mean 14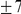 trimers. On the basis of an earlier data set of Yang et al [13] and the discretized 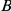-distribution, we estimated the stoichiometry of entry to be 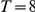 in the basic model [15]. We use this distribution and the respective estimate to demonstrate the estimation of the subunit stoichiometry with the basic model.

In their experiments, Yang et al. used HIV-1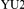 and HIV-1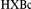 in 5 different experimental setups [19]. For the HIV-1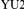 system, they performed three series of experiments. (i) Wildtype envelope proteins are coexpressed with D368R mutant envelope which makes the envelope CD4 binding defective. (ii) Wildtype envelope proteins are coexpressed with R315G/L317S. This mutation makes the envelope CCR5 binding defective. (iii) Wildtype envelope proteins are coexpressed with L520E mutant. This mutation introduces a charged residue in the normally hydrophobic peptide that disrupts membrane fusion.

For the HIV-1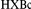, they studied two experimental setups with two different mutations. (iv) Wildtype envelope proteins are coexpressed with D368R mutated envelope protein. This mutation hinders CD4 binding. (v) Wildtype envelope proteins are coexpressed with R308L mutated envelope protein. This mutant is CXCR4 binding defective.

**Figure 7:**
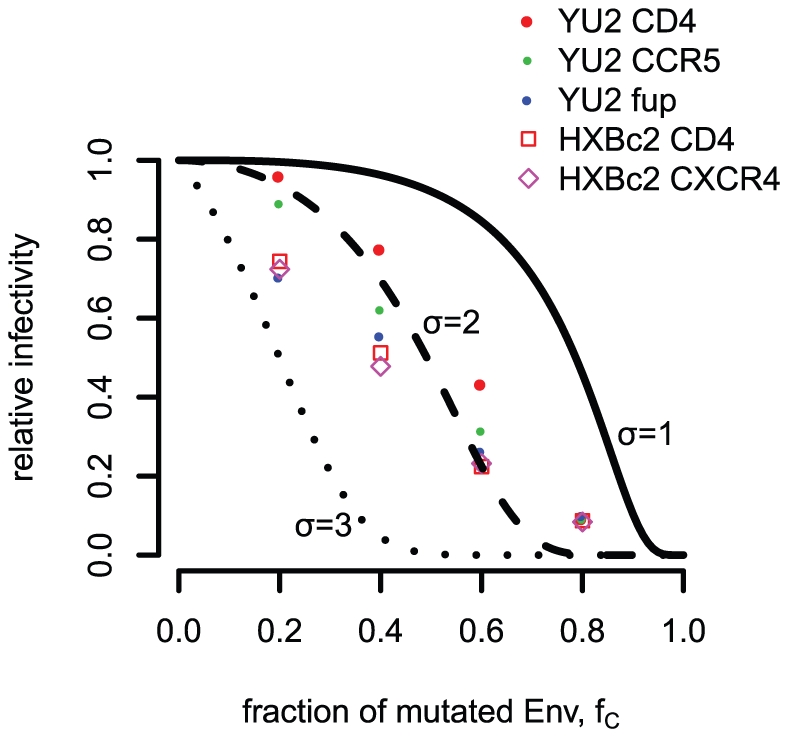
Predictions for the relative infectivity for different HIV-1 subunit stoichiometries using one type of mutated envelope protein. With this prediction the subunit stoichiometries for CD4- and coreceptor binding as well as for fusion proteins can be estimated. For this plot the basic model with stoichiometry of entry 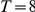 and the discretized 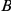-distributed trimer number are assumed.

Figure 7 shows the data points as well as the relative infectivities assuming the discretized 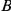-distributed trimer number and stoichiometry of entry 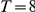. The relative infectivity for the HIV-1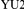 fusion proteins and the data for HIV-1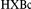 differ from those obtained for HIV-1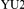. However, assuming the stoichiometry of entry 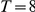 and the discretized 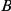-distributed trimer number with mean 14 and variance 49, the best estimate for the different subunit stoichiometries is two independent of the studied backbone and receptor. A bootstrap routine on the different data sets confirms these subunit stoichiometries; 100% of the bootstrap replicates result in an estimate of two for all the data sets except the fusion protein in the YU2 setting: 5.9% of the replicates give estimates of three and 94.1% of the replicates lead to an estimate of the subunit stoichiometry of two for this setup. This estimate of the subunit stoichiometries remain the same for 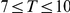, assuming the 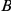-distributed trimer number with mean 14 and variance 49. The bootstrap routine for 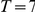 leads to higher uncertainties in the estimates for the YU2/fusion protein, the HXBc2/CD4 and the HXBc2/gen combinations (approximately 10% 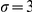 and 90% 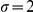). For 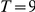 and 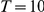 the estimates for the subunit stoichiometries are two in 100% of the replicates except for the YU2/CD4 combination (over 82% 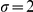, the rest 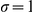). However, a bootstrap routine with only four data points per data set does not have strong statistical power. As Yang et al. did not use double mutants in their experiments nor combinations of wild-type envelope proteins and two envelope mutants, we cannot apply our framework for identifying potential subunit cooperation to this data.

## Discussion

In this paper we developed a mathematical framework to estimate subunit stoichiometries of viral spikes with a special focus on HIV trimers. The term subunit stoichiometry was formerly used to describe the number of envelope protomers that have to function to allow the whole trimer to take part in viral entry. We refine this term by studying the numbers of envelope proteins within one trimer that have to engage with CD4 receptors (CD4 subunit stoichiometry) and coreceptors (coreceptor subunit stoichiometry) as well as the number of fusion proteins that have to be revealed within one trimer such that this trimer takes part in mediating cell entry. With our models we identified two important input parameters that strongly influence the estimation of the subunit stoichiometries with infectivity assays using pseudotyped virions: The numbers of trimers on the different virions (the trimer number distribution) and the number of trimers that engage in mediating cell entry (stoichiometry of entry, [15]). Therefore, we strongly propose experimentalists to study the trimer number distribution and the stoichiometry of entry before estimating the subunit stoichiometries.

In our models, we predict the relative infectivities as functions of the stoichiometry of entry 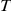, the trimer number distribution 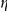, and the fraction of mutated envelope proteins 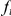 under the assumptions that

- the fraction of mutated envelope encoding plasmids 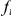 reflects the fraction of mutated envelope proteins in the virus producer cell from which trimers are sampled
- three envelope proteins are chosen perfectly randomly from the envelope pool to form a trimer, i.e. the number of mutated envelope proteins is Binomial distributed
- the trimers can move freely on the virion's surface and are recruited to the binding site
- the virion is infective if it has at least 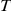 functional trimers

The models based on these assumptions are called *basic models*. Because none of these assumptions have been experimentally corroborated yet, we considered model extensions relaxing each of these assumptions in our studies of the stoichiometry of entry and neutralization [15], [17]:

- In the *imperfect transfection model* we allow the fraction of mutant envelope proteins in the envelope pool to differ from the fraction of mutant Env-encoding plasmids.
- For the *segregation model* we relax the assumption of binomial-distributed trimer assembly, i.e. the formation of trimers with only wild-type or mutant envelope proteins becomes more likely.
- In the *proximity model*, we assume that trimers have to be sufficiently close to each other for taking part in mediating cell entry.
- In the *soft threshold model* we relax the assumption of a strict thresholds for entry and scale the probability that a virion is infective with the number of trimers on its surface.

When fitting the imperfect transfection model and the segregation model to entry data [15], we obtain estimates for the imperfect transfection and the segregation model predicting that almost only homotrimers are expressed on the pseudotyped virions. The trimer tables in Figures 2 and 3 show that, in this case, neither the subunit stoichiometries nor the subunit cooperation could be estimated out of infectivity data with pseudotyped viruses. However, evidence for formation of mixed trimers was found in several studies [24], [27]. To fully understand stoichiometries in the context of virus entry and neutralization, it is therefore necessary to determine the degree of segregation or imperfect transfection with experiments rather than relying on simultaneous estimates of these parameters, already suggested in [15], [17]. The proximity model makes the assumption that trimers have fixed positions on the viral surface and cannot move. As the viral envelope stems from the cellular surface in which receptors can move freely, fixed trimer positions seem unlikely. The soft threshold model relaxes the assumption that virions that have fewer than 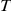 trimers cannot infect at all. The uncertainty in estimating the relevant parameters are extremely high. Therefore we only showed the model framework for the basic model in the present paper. However, as soon as more information on the imperfect transfection and segregation parameters, fixed trimer position and requirements for viral entry is available, the models for studying the subunit stoichiometries can be extended accordingly. To this end, the probability to form a functional trimer must be adjusted following the lines presented in [15], [17].

In addition, we describe an experimental setup and the corresponding mathematical models to test whether two functions have to be located on the same envelope protein. In the case of HIV-1, one can address with this framework whether (i) the CD4 receptor and the coreceptor must bind to the same envelope protein, (ii) the fusion protein of the same envelope protein is revealed as the CD4 receptor has bound to or (iii) the fusion protein of the same envelope protein is revealed as the coreceptor has bound to. We demonstrate which mutations have to be used in infectivity assays with pseudotyped virions to obtain signals that allow to determine the two binding scenarios in a general setting dependent on the values for the single subunit stoichiometries. This model extension is inspired by the HIV trimer to which CD4 and coreceptors must bind for cell entry. Conformational changes induced by CD4 binding make the coreceptor binding possible [28]. These studies are performed for monomers [21], [29], [30]. The possibility that binding of one CD4 receptor to one envelope protein within the trimer induces also conformational changes in the neighboring envelope proteins has not been ruled out. Therefore, it might be possible that coreceptor binding might happen at a different envelope protein, e.g. due to steric hindrance. The framework we developed for testing this potential cooperation identifies which experiments have to be done when the actual values for the subunit stoichiometries are finally determined. In the specific case of the subunit stoichiometries being two, an experimental system with two different envelope mutants and the envelope wildtype must be employed. The experimental work for such systems would be enormous if one wants to test all possible pseudotyped viral stocks. With our framework we showed, that only a small number of experiments are already sufficient for identifying subunit cooperation.

The concept of subunit stoichiometries might be also helpful for other viruses that enter host cells via binding of their surface proteins to more than one host cell receptor. We will illustrate how our models can be extended with the following two examples:

The haemagglutinin (HA) of Influenza virus is the viral spike that binds the viruses to cell-surface glycoconjugates and after endocytosis it mediates fusion of the viral and endosomal membrane. As the HIV-1 spike, HA consists of there identical subunits, each being a dimer of two polypeptides HA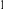 and HA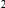 [31]. The experimental system for studying the HA subunit stoichiometries must consist of a pseudo-typed viral system with HA subunits resistant against glycoconjugate binding and mediating fusion in the endosome. Infectivity experiments with these pseudo-typed virions can be used to estimate the number of glycoconjugates that have to bind to the HA and the number of fusion domains that have to interact for mediating membrane fusion. In addition, by using the concept of co-function subunit stoichiometries, one can also find out which of the HA subunits must bind to the glycoconjugates and reveal the fusion domain.

The other example considers the hepatitis C Virus (HCV) which enters its target cells via endocytosis but attaches first to glycosaminoglycans (GAG) and CD81 on the target cell surface [32]. The spikes that establish contact to those receptors consist of two envelope glycoproteins E1 and E2 assembling as non-covalent dimers [33]. By using an in vitro cell infection system in combination with envelope variants resistant against GAG or CD8, one can study how many of these receptors have to bind to the dimer and how this receptor binding interacts.

The caveats of our suggested method are mostly limitations in the experimental system. In vitro infectivity assays can only be performed with viruses for which infectable cell lines exist. In addition, the viruses must be genetically engineered with the technique of pseudotyping viruses. For the envelope proteins, mutations are needed that confer resistance to receptor binding or that have defective fusion proteins. These envelopes must also be integrated into the spike along with the wild-type variants of the envelope protein.

For HIV-1, Yang et al. [19] already performed infectivity experiments with pseudo-typed viruses expressing mixed trimers of wild-type and mutant envelope proteins. Several different mutants were used that made one envelope CD4-binding deficient, coreceptor-binding deficient and had defects in the fusion proteins. The model they used for analyzing the data did not include the variation in trimer numbers on the surface of the HIV-1 virions. In addition, they did not distinguish between different subunit stoichiometries nor did they use double mutants. We re-analyzed their data with our models allowing for different subunit stoichiometries for the CD4 binding, the coreceptor binding and the fusion protein. Although the data might suggest that the subunit stoichiometries vary between receptor binding and fusion protein revelation in the YU2-system and for receptor binding between the YU2 and the HxBc2 system, this difference does not lead to different estimates for the subunit stoichiometries. Louder et al. [34] showed that pseudotyped virions might have a lower expression level of trimers on their surface. The expression levels between YU2 and HxBc2 might be different leading to different trimer number distributions for the two systems. As we have shown in Figure 4C different trimer number distributions lead to different predictions for the subunit stoichiometries and might be the reason for the differences between the YU2 and HxBc2 data for CD4 and the coreceptor. The difference between the CD4/coreceptor and fusion protein stoichiometries in the YU2 data might be due to different levels of segregation. As we argued earlier, possible segregation should be studied in a separate line of experiments and could be integrated into our models.

Liu et al. [24] performed infectivity assays with pseudotyped virions. The envelope proteins used in their experiments were either CD4 binding defective or had defective fusion proteins. If one assumed that both subunit stoichiometries were two, these pseudotyped virions should not be infective at all. Whilst the efficiency of infection decreased approximately 100-fold, infection with these virions could still be observed. These observations make it impossible to rule out the possibility that the CD4 and fusion subunit stoichiometry are less than two.

The concept of stoichiometries [12]–[17] allows us to study the requirements for entry and neutralization from the virion's perspective. But the host cell also has to fulfill certain conditions for being infected. In the case of HIV, CD4 receptors and coreceptors must be expressed on the cellular surface for successful infection of the cell. Individuals having a homozygous defect in the CKR-5 gene, the gene encoding for the CCR5 receptor, are rarely infected with HIV upon repeated HIV exposures and CD4 T-cells of such individuals must be challenged with a 1000-times higher viral dose to be infected in vitro [35]. Recently, it was shown, that there is a correlation between expressed cellular receptors and infectability by HIV [36], [37]. Earlier experiments showed that cells expressing a high number of CD4 receptors needed a lower expression of CCR5 receptors for being infected with HIV virions. Vice versa, cells with a low number of CD4 receptors needed a higher number of CCR5 receptors for infection with the same HIV strain [38]. For infectivity assays with pseudotyped virions, the target cells are assumed to express a sufficiently high number of CD4 and CCR5 receptors to guarantee that every possible HIV binding site can engage with a receptor. Only under this condition it is guaranteed that host cell requirements do not influence the estimation of the subunit stoichiometries. However, combining the stoichiometry of entry with the subunit stoichiometries, we can calculate the minimal number of CD4 receptors and coreceptors that are required for entry of a complete virion. Due to stochastic and steric effects, this number is only a lower bound for the number of cellular receptors that makes a cell infectable. In the future, it would be interesting to study subunit stoichiometries with target cells that differ in their CD4 and CCR5 expression levels. These experiments will additionally inform about the host cell requirements.
